# Salvianolic acid B regulates gene expression and promotes cell viability in chondrocytes

**DOI:** 10.1111/jcmm.13104

**Published:** 2017-02-28

**Authors:** Xiaohong Yang, Shaojie Liu, Siming Li, Pengzhen Wang, Weicong Zhu, Peihong Liang, Jianrong Tan, Shuliang Cui

**Affiliations:** ^1^ Guangzhou Institute of Traumatic Surgery Guangzhou Red Cross Hospital Jinan University School of Medicine Guangzhou China; ^2^ Department of General Surgery Guangzhou Red Cross Hospital Jinan University School of Medicine Guangzhou China; ^3^ Department of Zoology Faculty of Science the University of Melbourne Parkville Victoria Australia

**Keywords:** salvianolic acid B, β‐catenin, *Cytl‐1*, *Sox9*, collagen type II (COL II), collagen type I (COL I), chondrocyte activity, viable cells, passaged chondrocytes

## Abstract

Articular chondrocytes reside in lacunae distributed in cartilage responsible for the remodelling of the tissue with limited ability of damage repairing. The *in vitro* expanded chondrocytes enhanced by factors/agents to obtain large numbers of cells with strengthened phenotype are essential for successful repair of cartilage lesions by clinical cell implantation therapies. Because the salvianolic acid B (Sal B), a major hydrophilic therapeutic agent isolated from *Salvia miltiorrhiza*, has been widely used to treat diseases and able to stimulate activity of cells, this study examines the effects of Sal B on passaged chondrocytes. Chondrocytes were treated with various concentrations of Sal B in monolayer culture, their morphological properties and changes, and mitochondrial membrane potential were analysed using microscopic analyses, including cellular biochemical staining and confocal laser scanning microscopy. The proteins were quantified by BCA and Western blotting, and the transcription of genes was detected by qRT‐PCR. The passaged chondrocytes treated with Sal B showed strengthened cellular synthesis and stabilized mitochondrial membrane potential with upregulated expression of the marker genes for chondrocyte phenotype, *Col2‐α1*,* Acan* and *Sox9*, the key Wnt signalling molecule *β‐catenin* and paracrine cytokine *Cytl‐1*. The treatments using CYTL‐1 protein significantly increased expression of *Col2‐α1* and *Acan* with no effect on *Sox9*, indicating the paracrine cytokine acts on chondrocytes independent of SOX9. Sal B has ultimately promoted cell growth and enhanced chondrocyte phenotype. The chondrocytes treated with pharmaceutical agent and cytokine in the formulated medium for generating large number of differentiated chondrocytes would facilitate the cell‐based therapies for cartilage repair.

## Introduction

The physiological development and remodelling, and the repair of defects of the articular cartilage require viable chondrocytes with interactive activities, on which cartilage repair processes depend, particularly for cell‐based therapeutic strategies, such as autologous chondrocyte implantation (ACI) 1, 2. The large enough numbers of chondrocytes have to be obtained by *in vitro* cultivation of cells isolated from cartilage of limited biopsy sampling 1, 3, 4 and retain their differentiation status 5, 6, 7. Chondrocytes are the only type of residential cells in the avascular, aneural and alymphatic load‐bearing cartilage 8, 9, interacting with the extracellular matrix (ECM) to sense the mechanical and biological stimulations and respond by cellular secretions and signal transduction for continually remodelling and damage repair 10, 11. Cartilage development and remodelling is regulated by coordinated chondrocytic activities through cellular pathways such as Wnt/β‐catenin signalling 12, 13, 14 in conjunction with growth/transcription factors *i.e*. SOX9 15, 16, 17 and cytokines *i.e*. the chondrocyte‐specific novel cytokine‐like factor CYTL‐1 18, 19. The interplay between SOX9, cytokines and Wnt/β‐catenin signalling in chondrocytes dynamically balance the proliferation and differentiation 16. Active chondrocytes are essential for clinical treatments to regenerate cartilage and restore articular function 20, 21, and the cellular functions can be promoted by signalling/growth factors 22, 23 and medicinal additives 24, 25, which usually act *via* regulating biofactors. The enhanced cells and their bioactivity would certainly be beneficial to the treatments for repair of damaged cartilage. Medicinal molecules acting on chondrocytes to regulate their activity are of great importance in modulating the process of cartilage regeneration.

The *Salvia miltiorrhiza* (SM), a traditional Chinese medicine herb, has been used either as extracts or as isolated individual components for treating a great range of diseases in traditional and modern medicine 26, 27. The SM components were applied in medical treatments for various diseases and revealed cellular and molecular pathways in which SM exerts its effects on cells and tissues 28, 29. SM extracts were also employed in treatments of skeletal diseases such as osteoporosis through targeting specific pathways in bone resorption and bone formation 30, 31. Salvianolic acid B (Sal B), a hydrophilic component of SM, was reported to act on variety of cell types to regulate cellular activities 32, 33, 34, including osteogenesis 35, 36. Little is known about actions of SM and its components on chondrocytes potentially applicable in therapeutic approaches for cartilage regeneration.

This study provided evidence showing the biological actions of Sal B on cultured chondrocytes. Sal B treatments demonstrated enhanced anabolic activity in the chondrocytes by elevating mitochondrial membrane potential and stimulated cell survival and synthetic activity exhibited as increased volumes of nucleic acids by specific labelling and quantitative analysis. Molecular analyses of chondrocyte‐specific gene expression found upregulated transcription of genes encoding chondrocytic proteins for cartilage along with genes encoding key regulator and transcription factor for regulation of cell growth. The expression regulation of these genes seemed to be in a similar manner of dosage effect. The upregulation of some of those genes was also exhibited at protein level as analysed comparatively by total cellular proteins and specific proteins determined by Western blots. Further study demonstrated that CYTL‐1 increased the expression of genes for chondrocyte phenotype but no effect on SOX9, which indicates that Sal B directly stimulated the expression of SOX9 rather through CYTL‐1. The viability and the chondrocytic phenotype of treated cells were ultimately enhanced in a dosage effective manner within the testing period of cell proliferation. These promoted cellular activities and increased viable chondrocytes by Sal B would be essentially beneficial and applicable to treatments for osteochondral damage repairs.

## Materials and methods

### Isolation and culture of primary chondrocytes

Rabbit cartilage obtained from articular surfaces was minced and sequentially digested by the following *Sigma* enzymes, 0.05% hyaluronidase, 0.25% trypsin and 0.4% collagenase to harvest primary chondrocytes for monolayer culture, as described in PROTOCOL 22.16 37. The isolated cells were washed and suspended in DMEM‐F12 complemented with 15% FBS (GIBCO/Life Technology, NY, USA) and 1.0% penicillin‐streptomycin solution. Prepared primary chondrocytes were seeded in 25‐cm^2^ flasks with 8 × 10^5^/ml cells and cultured in DMEM‐F12 medium till 80% confluence followed by subculture for obtaining enough cells at about passage 3/4, termed Amplified Cells as starting material for experiments. About 1.7 × 10^4^ cells of Amplified Cells were applied onto a 24 × 24 mm coverslip and cultured for 24 hrs in DMEM‐F12 and then cultured in the medium containing Sal B (MW = 718.614, National Institute for Food and Drug Control, Beijing, China) for 24 hrs. The cells were harvested and fixed with 4.0% paraformaldehyde to make the Fixed Cells on Coverslip.

### Immunohistochemical staining of COLs

Fixed Cells on Coverslip were washed with their endogenous peroxidases deactivated using 3% H_2_O_2_ and then used for detection of collagen type I (COL I) and collagen type II (COL II) by the Immunohistochemical StreptAvidin‐Biotin Complex (SABC SA1027; Boster, Wuhan, China) according to the manufacturer's instruction (SA1027; Boster, Wuhan, China). Cells were incubated overnight with 1:200 diluted antibodies of COL1A (COL‐1) and COL2A1 (Santa Cruz, CA, USA) against COL I and COL II, respectively, and the binding proteins were visualized by colorimetric reaction between the bound biotinylated rabbit anti‐goat IgG antibody and diaminobenzidine, which was fixed by dehydration, cleared with xylene and sealed using DPX Mountant (VWR BDH, Radnor, PA, USA) for microscopic analysis.

### Toluidine blue staining of proteoglycan

Fixed Cells on Coverslip were washed in PBS and stained in toluidine blue solution (Sigma‐Aldrich, St. Louis, MO, USA) for 20 min. The stained cells were quickly dehydrated through a 95% and two changes in 100% ethanol (10 dips in each step as the stain fades in ethanol), cleared with xylene and sealed using DPX Mountant for microscopic analysis.

### Scanning electron microscopy (SEM)

About 1.7 × 10^4^ Amplified Cells were cultured for 24 hrs and then continued for 24 hrs in the established medium containing 14.0 μM Sal B for treatments with an equivalent amount of 1× phosphate‐buffered saline (PBS) for controls. Then, the cells were fixed on coverslips for 24 hrs in 2.5% glutaraldehyde in 1× PBS and analysed by scanning electron microscopy (XL‐30‐based Environmental Scanning Electron Microscope, Philips, Hilversum, Netherlands).

### Detection of mitochondrial membrane potential

About 1.7 × 10^4^/ml Amplified Cells were incubated for 24 hrs, and then the alive cells were placed in a self‐designed device resembling the Nunc Glass Bottom Cell Culture Dishes (Thermo Scientific, Waltham, MA, USA). Drops of 10 μg/ml hydrochlorothiazide rhodamine 123 (HR 123; Sigma‐Aldrich) solution was added to the cells and incubated at 35–37°C for 10 min. for tracking the mitochondrial activity. Selected region of interest (ROI) for both experimental and control cells was observed for 5 min. to obtain a baseline of label intensity; then, 14.0 μM Sal B was applied to the experimental cells with equal volume of 1× PBS used for the control cells. Sequential images recording the dynamic changes of the cellular mitochondrial membrane potential shown as fluorescent intensities (indicated by pseudo‐colour code) were acquired for 25 min. Selected images from the sequence in Sal B‐treated cells were compared to their counterparts from the control cells.

### Acridine orange labelling of nucleic acids

Fixed Cells on Coverslip were washed and stained for 10 min. using 0.01% acridine orange (Sigma‐Aldrich) solution to specifically generate green emission for DNA and red for RNA. Images of stained cells were viewed and captured using confocal laser scanning microscopy (CLSM, Zeiss LSM 510 META System, Jena, Germany). The green and red labels in the cell content acquired by the dual channels were quantitatively analysed using the Zeiss Physiology/TimeSeries for Release 3.2.

### Quantitative analysis of gene expression by qRT‐PCR

About 6.3 × 10^5^ Amplified Cells were transferred to 25‐cm^2^ flasks (final total volume of 10 ml), treated with 28.0 μM Sal B in culture for 24 hrs. The total RNA was prepared using TRIzol reagent (Invitrogen, Carlsbad, CA, USA) from the chondrocytes and converted to cDNA using PrimeScript^®^RT Master Mix (TaKaRa, Shiga, Japan). The qRT‐PCR was performed in triplicate using gene‐specific primers (Table 1) and the All‐in‐One qPCR Mix (GeneCopoeia) with SYBR Green labels (Thermo Scientific, Rockville, MD, USA) according to the providers’ protocols. The house‐keeping gene Glyceraldehyde 3‐phosphate dehydrogenase (GAPDH) was used as an internal control, to which the abundance of target gene expression was normalized.

**Table 1:** Primers designed for qRT‐PCR analysis in rabbit chondrocytes

Gene name	Accession No.	Forward primer (5′ – 3′)	Primer size	Reverse primer (5′ – 3′)	Primer size	Amplicon size
*Acan*	XM_002723376	GGATGGACACCCCCTACAA	19	AGGGGACGTCATTCCACTC	19	122
*Axin‐1*	XM_005255607	TCGAGGGCGAGAAGGAGATC	20	GGGCGTTCAATGGACAAGG	19	109
*Col1‐α1*	XM_017348831.1	TGCCCAGAAGAACTGGTACA	20	AAGCCATCGGTCATGCTCTC	20	81
*Col2‐α1*	NM_001195671	GACGACATAATCTGTGAAGACACC	24	GTTCTCCTTTCTGCCCCTTTG	21	133
*Cytl‐1*	XM_002708369	AGGAGGGATTTGGTGTTC	18	CATTCTCTGGCATGAAGC	18	95
*Dvl‐1*	AAGW02049594^a^	GGACGTGGTCGACTGGCTC	19	GTGATCTTGTTCACGGTGTGCC	22	120
*GAPDH*	NM_001082253	AGGGCTGCTTTTAACTCTGG	20	ATGACCAGCTTCCCGTTCT	19	149
*Gsk‐3β*	XM_002716660	TAATCAAGGTCCTGGGAACAC	21	CGCACTCCTGAGGTGAAA	18	142
*Hif‐1α*	NM_001082782	GACTTCCAGTTGCGGTCCTTCG	22	TTTAATCGTCAGTGGTGGCGGT	22	141
*IL‐1β*	NM_001082201.1	GGTGTTGTCTGGCACGTATG	20	GGCCACAGGTATCTTGTCGT	20	124
*Sox9*	XM_002719499	CTGGAGACTGCTGAACGAGAG	21	GGTACTTGTAGTCCGGGTGGT	21	98

a Oryctolagus cuniculus cont2.49593, the whole genome shotgun sequence was used for designing of rabbit *Dvl‐1* primers.

### Regulation of CYTL‐1 on gene transcription in chondrocytes

About 1.5 × 10^5^ of Amplified Cells were allocated to make a final volume of 2.0 ml in wells of 12‐well plate and cultured in the medium containing diluted CYTL‐1 protein for 24 hrs at 37°C with 5.0% CO_2_ supply in DMEM‐F12 medium containing 4.0, 8.0, 16.0, 32.0 μM of purified recombinant protein of Homo sapiens cytokine‐like 1 (rhCYTL‐1, Cat#: TP306778; OriGene Technologies Inc., Rockville MD, USA) and equal volumes of 1× PBS for control cells. The cells were harvested for total RNA isolation, cDNA synthesis and qRT‐PCR analysis as described in ‘Quantitative analysis of gene expression by qRT‐PCR.’

### Protein analyses

The total proteins were prepared from cultured chondrocytes obtained as in ‘Quantitative analysis of gene expression by qRT‐PCR’ by lysis in the suspension of the RIPA Lysis and Extraction Buffer (Thermo Scientific) using Sonics Vibra Cell™ (Sonics & Materials, Newtown, CT, USA). Total cellular proteins were quantitatively analysed using the bicinchoninic acid (BCA) total protein quantitation assays 38. Equal proteins were separated by 8.0% SDS‐PAGE, blotted onto nitrocellulose membrane (Bio‐Rad, Hercules, CA, USA). The membranes were blocked by skim milk powder solution in Tris‐buffered saline and then incubated with diluted primary antibodies of CTNNB1 (AVIVA, San Diego, CA, USA) against β‐catenin, COL2A1 (Santa Cruz) against COL II by specifically binding to the C‐terminal epitope of a highly conserved motif between human and rabbit, and COL1A (COL‐1; Santa Cruz) against COL I, respectively, with the Monoclonal Anti‐β‐Actin (Sigma‐Aldrich) against the house‐keeping gene β‐actin used as an internal control. The binding proteins were detected with conjugated secondary antibody, goat anti‐rabbit IgG‐HRP (Santa Cruz) and visualized using Clarity™ Western ECL Substrate Kit (Bio‐Rad). The images were captured and analysed using the Image Lab (Beta 1) Version 3.0.1 Changelist 40296 software associated with Molecular Imager^®^, ChemiDoc™ XRS+ Imaging System (Bio‐Rad).

### Detection of the effect of Sal B on chondrocyte proliferation

About 5 × 10^3^ of Amplified Cells were transferred to 24‐well plate to make final volumes of 1.0 ml/well, further cultured in the medium complemented with 4.34, 8.75, 17.5, 35.0, 70.0 and 140.0 μM Sal B and equal volumes of 1× PBS in control cells for 24 hrs or cultured in the medium containing selected Sal B concentrations of 7.0, 14.0, 28.0 μM for 8 days and media were changed every second day and complemented with freshly dissolved Sal B for the long period experiments. The cell viability was assayed by MTS using CellTiter 96^®^ AQueous One Solution according to the manufacturer's instruction (Promega, Madison, WI, USA) and documented by their GloMax Multi Detection System.

### Statistical analysis

Differences of OD values and tendency of interactive effects of two factors between Sal B‐treated and control chondrocytes cultured in eight consecutive days were analysed using two‐factor repeated‐measures analysis of variance (anova) in SAS 9.3 (SAS Institute Inc., Cary, NC, USA). Other differences between groups were analysed by one‐way anova using SPSS 19 (IBM SPSS Statistics, Chicago, IL, USA). Differences with a *P* value of <0.05 were considered significant (*), and the *P* value of <0.01 were regarded as highly significant (**).

## Results

### Culture and characterization of rabbit chondrocytes

**Figure 1:**
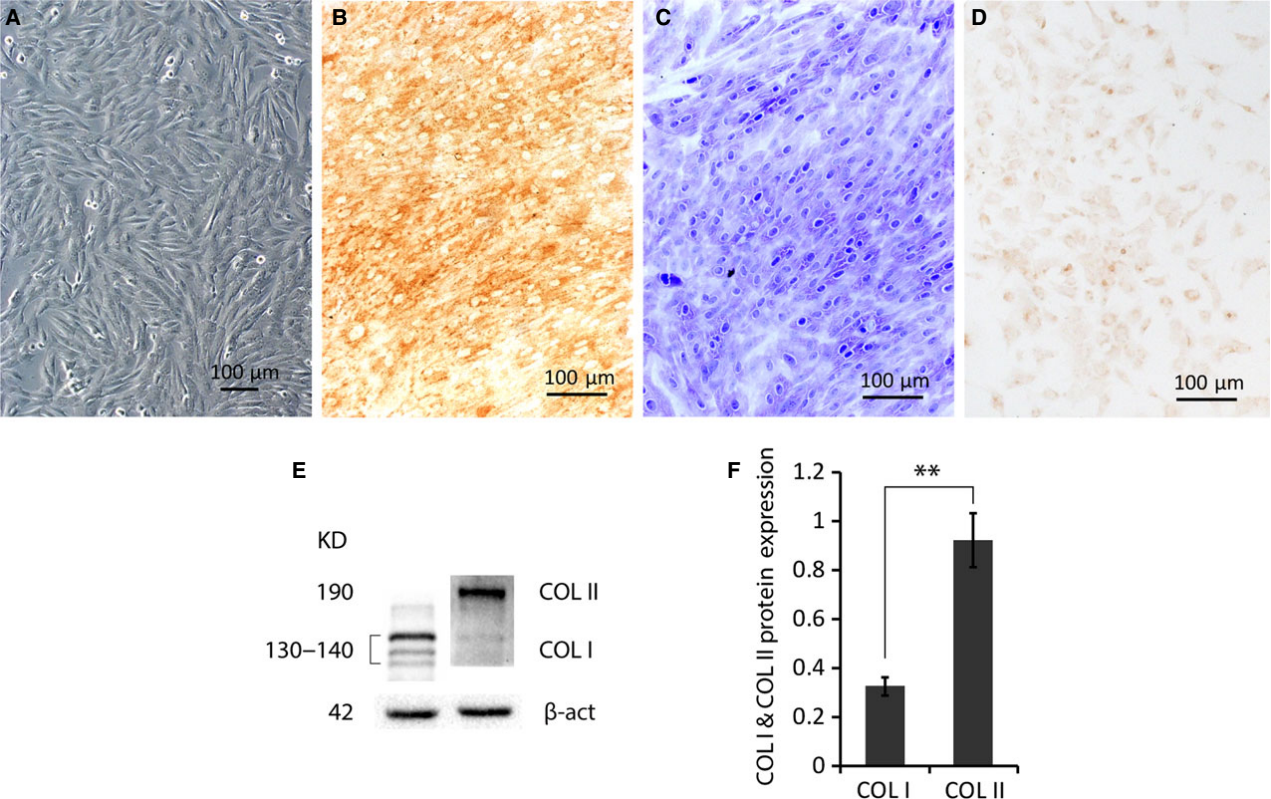
Characterization of cultured rabbit chondrocytes. (**A**) A typical colony morphology of the passage 3 chondrocytes showed by inverted phase contrast microscopy. (**B**) Chondrocytic protein COL II and (**D**) COL I in cultured cells were exhibited by immunohistochemical staining using specific antibodies, respectively. (**C**) Cellular proteoglycans were specifically stained by toluidine blue. (**E**) Representative Western blotting of COL I and COL II in total proteins of cultured chondrocytes using specific antibodies. (**F**) The plotting (*n* = 4) of normalized densities against the internal control β‐actin shows much higher COL II (≈3 times) than COL I (*n* = 4). **P < 0.01.

The isolated chondrocytes in culture exhibited spherical format when first inoculated in the medium, then adhered and fused to form monolayers, grown confluent (95%) within 3 days. The monolayer of confluent cells showed typical swirling fibrous colonies after passage 3 (Fig. 1A), and predominantly expressed COL II (Fig. 1B) and secreted proteoglycan (Fig. 1C) as a typical chondrocytic phenotype with a certain level of COL I expression (Fig. 1D) as a characteristic of dedifferentiation. The cultured chondrocytes expressed both COL I and COL II proteins (Fig. 1E) as detected by Western blotting (*n* = 4), but about three times of COL II expressed (*P* < 0.01) compared to COL I expression (Fig. 1F).

### Enhanced chondrocyte morphology by SEM

**Figure 2:**
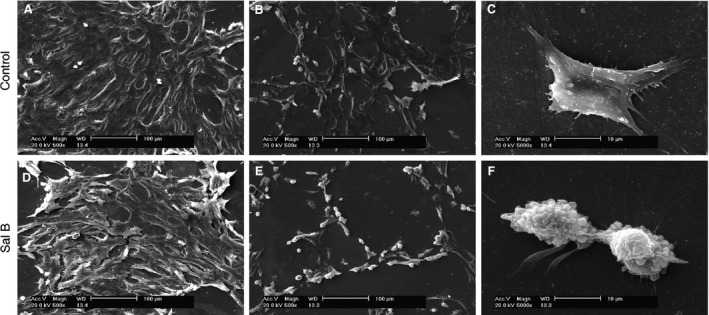
Morphological details of chondrocytes revealed by scanning electron microscopy (SEM). (**A**–**C**) Colony morphology of untreated cells. (**D**–**F**) Morphology of chondrocytes treated with 14.0 μM Sal B for 48 hrs. Compared to untreated cells, the treated chondrocytes exhibit stimulated cellular properties, presented as dense aggregates liaised with active cells and typically shaped chondrocytes with extruded granules surrounded by secreting filamentous. Scaled bar (﹣) represents 100 μm in (**A**), (**B**), (**D**) and (**E**); 10 μm in (**C**) and (**F**).

Chondrocytes were less condensed in aggregates (Fig. 2A) with active cells scattered (Fig. 2B) in spreading flat areas of irregular shapes (Fig. 2C) in control groups, which seems less active than Sal B‐treated chondrocytes formed dense aggregates surrounded by active cells (Fig. 2D), tandemly expanded to make connections between aggregates (Fig. 2E), in triangle‐like shapes with a large number of extruded granules predominantly visible on the cell surface under high magnification (Fig. 2F).

### Enhanced nucleic acid synthesis in treated chondrocytes

**Figure 3:**
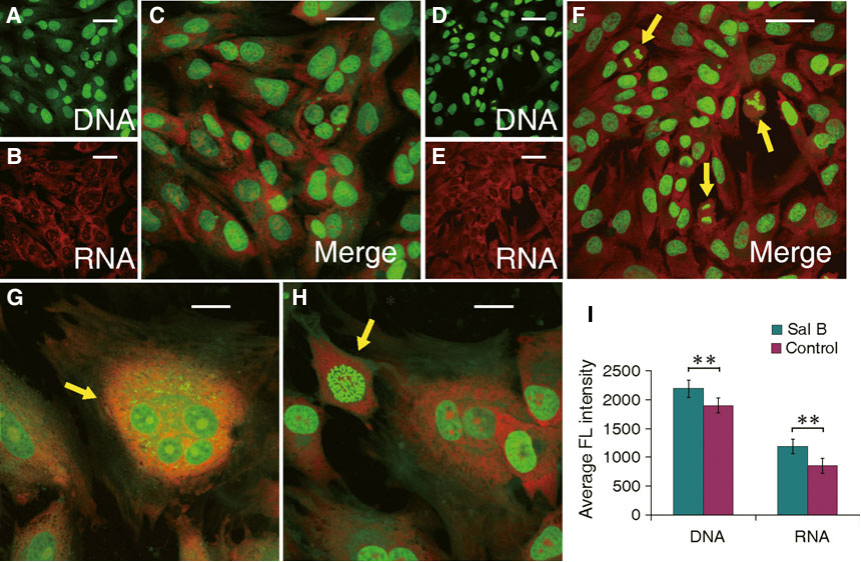
Morphological changes of Sal B‐treated chondrocytes analysed by acridine orange staining and CLSM. The specific intercalary labelled DNA and electrostatic attractively labelled RNA in cells (cationic) excite and emit at different wavelength to be captured as fluorescent green and red, respectively. The labelled nucleic acids display the contour of cells, and their relative intensity implicates DNA duplication and RNA synthesis. (**A**–**C**) The acquired images of chondrocytes with stained DNA (**A**) and RNA (**B**) merged into dual‐channel image (**C**) in untreated cells. (**D**–**H**) The acquired images of chondrocytes with labelled DNA (**D**) and RNA (**E**) images then merged to a dual‐channel image (**F**) for Sal B‐treated cells. Higher level of DNA and RNA staining is apparent in Sal B‐treated chondrocytes (**F**) than those in control cells (**C**), which reflect the promoted genetic processing and gene transcription and were further showed in various subcellular formats (indicated by arrows) in the cycling cells (**F**–**H**). (**I**) Significant higher levels of DNA and RNA in Sal B‐treated chondrocytes (green) than those in control cells (purple) were quantitatively revealed by intensity analysis of the dual fluorescent labelling. Scaled bar (﹣) represents 50 μm. **P < 0.01.

Acridine orange stained chondrocytes through interaction with DNA and RNA, which were showed in green and red, respectively, according to their specific excitation and emission wavelengths. The nucleic acid staining of Sal B‐treated cells outlined the cellular structures of different cycling phases by presenting typical multi‐shaped chondrocytes with, sometimes, double‐ and poly‐nuclei cells, duplicating chromosomes and cell aggregates in contrast to their control counterparts (Fig. 3A–H) by CLSM. Quantitative analysis of labelled nucleic acid in Sal B‐treated chondrocytes revealed a significantly higher level (*P* < 0.01) in both DNA and RNA contents (Fig. 3I) than untreated cells.

**Figure 4:**
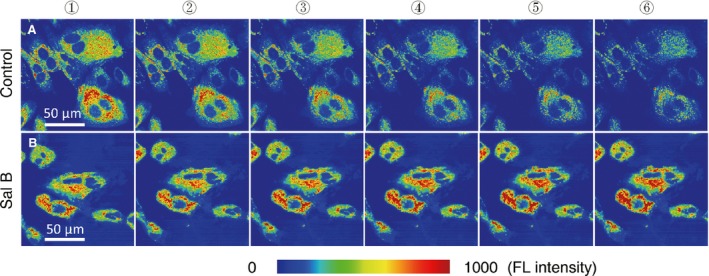
Retention of stimulated mitochondrial membrane potential in alive chondrocytes treated by Sal B. Series of images from the region of interest (ROI) were acquired by CLSM for 25 min. after the cells were stained with HR 123, which specifically labels mitochondria with fluorescence colours presented as fluorescence intensity of blue (weak) gradually to red (strong). (**A**) Control cells: the labelled mitochondria in ROIs before the Sal B treatment presented as the baseline (**A**①), then images acquired and selected by 5 min. intervals (**A**②–⑥). The fluorescence labels for active mitochondria faded away as the time lapses. (**B**) Sal B‐treated cells: the labelled mitochondria in ROIs before Sal B stimulation set as the baseline (**B**①), followed by images acquired and presented as in (**A**) (**B**②–⑥). The stimulated mitochondrial membrane potential in the treated chondrocytes holds during the experiment with a trend of increasing.

The mitochondrial membrane potential of chondrocytes was examined by HR 123 staining and expressed as changes of the fluorescent label intensity, which was detected and recorded by CLSM. The signals were recorded for 25 min. as measurements of the mitochondrial membrane potential. Untreated chondrocytes started to show the baseline (Fig. 4A①), then faded away gradually (Fig. 4A②–⑥), while the treated chondrocytes maintained the baseline steadily with an increment throughout the testing period (Fig. 4B①–⑥).

### Modulated protein and gene expression by Sal B in chondrocytes

**Figure 5:**
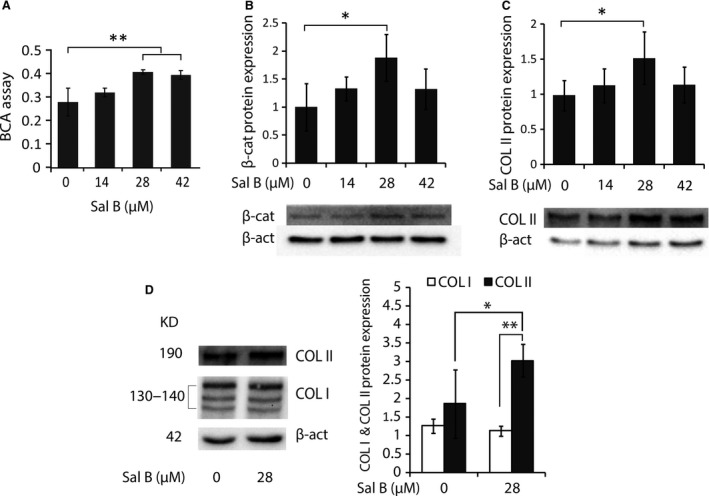
Protein analyses in cultured chondrocytes. About 6.3 × 10^5^ Amplified Cells were cultured in 10 ml of medium with or without Sal B for 24 hrs and the cultured chondrocytes were used for preparation of total proteins. The samples analysed by BCA assays and Western blotting using specific antibodies (Materials and Methods). (**A**) BCA assays revealed significantly increased total cellular proteins in chondrocytes treated with 28.0 and 42.0 μM Sal B. (**B**) Western blot analysis using the antibody CTNNB1 found a significantly increased β‐catenin level in chondrocytes treated with 28.0 μM Sal B. (**C**) The COL II protein level was also significantly elevated in chondrocytes treated with 28.0 μM Sal B as detected by Western blot using the antibody COL2A1. (**D**) When chondrocytes in culture were stimulated using the effective concentration of 28.0 μM Sal B, Western blots using the antibody COL1A and COL2A1 showed that COL II was significantly higher than COL II in untreated cells (*) and COL I in both treated and control cells (**). The level of COL I remained unchanged. The house‐keeping protein β‐actin (β‐act) was used as an internal control in the analyses. *n* = 4, *P < 0.05 and **P < 0.01.

**Figure 6:**
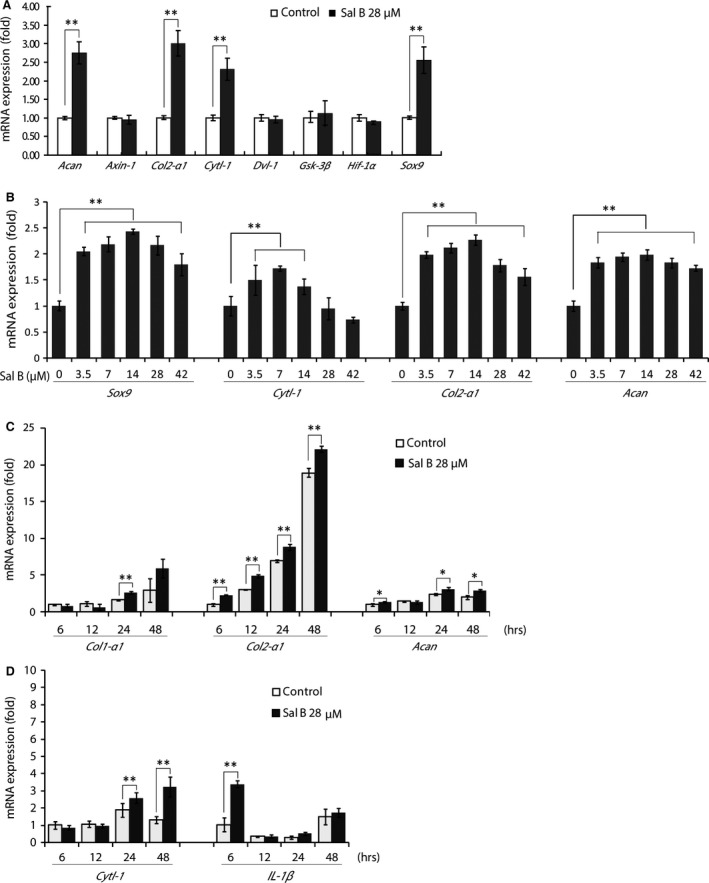
qRT‐PCR analyses of gene transcription. (**A**) Unchanged expression of genes coding for key regulators involved in Wnt/β‐catenin signalling pathway (*Axin‐1*,* β‐cat*,* Dvl‐1*,* Gsk‐3β* and *Hif‐1α*) and upregulated expression of genes coding for transcription factor SOX9 (*Sox9*), the cytokine CYTL‐1(*Cytl‐1*) and genes coding for chondrocyte proteins of collagen type II (*Col2‐α1*) and Aggrecan (*Acan*) in 28.0 μM Sal B‐treated chondrocytes. (**B**) qRT‐PCR analyses demonstrate actions of various concentrations of Sal B on expression of *Sox9*,* Cytl‐1*,* Col2‐α1* and *Acan* in cultured chondrocytes in a similar dosage effective fashion, by which the expression of these genes was significantly upregulated by a series of dilutions of Sal B accept the high concentrations of 28.0 and 42.0 μM for *Cytl‐1*. (**C**) The expression of *Col1‐α1*,* Col2‐α1* and *Acan* in chondrocytes treated with 28.0 μM Sal B in a time course of 6, 12, 24 and 48 hrs was investigated by qRT‐PCR. The expression of *Col2‐α1* and *Acan* was remained upregulated in the testing period accept 12 hrs for *Acan*, while *Col1‐α1* was not stimulated by Sal B in the analysis even using six times cDNA templates beside an increase at 24 hrs. (**D**) The time course study for the expression of cytokine‐like factor *Cytl‐1* and interleukin‐1β (*IL‐1β*). The *IL‐1β* was highly upregulated by Sal B treatment at 6 hrs and then became unchanged, while the *Cytl‐1* expression remained unchanged in the early hours and upregulated at 24 hrs and 48 hrs. The house‐keeping gene GAPDH was used as an internal reference. *P < 0.05 and **P < 0.01.

The total proteins in all Sal B‐stimulated chondrocytes were increased in a dosage‐dependent manner with a maximum mount (*P* < 0.01) detected in the cells treated with 28.0 and 42.0 μM Sal B (Fig. 5A). The key Wnt signalling factor β‐catenin and the phenotypic protein COL II were significantly increased (*P* < 0.05) in cultured chondrocytes treated with 28.0 μM Sal B (Fig. 5B and C) as detected by quantitative Western blot analysis. Further investigations showed expression of both COL I and COL II proteins (Fig. 5D) in Sal B‐treated chondrocytes, but the level of COL I remained unchanged, while the COL II level in treated cells was significantly higher than COL I in both groups (*P* < 0.01) and COL II in untreated cells (*P* < 0.05). The qRT‐PCR analysis revealed a significant upregulation (*P* < 0.01) of the master transcription factor *Sox9*, the important autocrine/paracrine chondrocytic cytokine *Cytl‐1* and the genes of *Col2‐α1* and *Acan* coding for the major downstream products of COL II and Aggrecan (ACAN) in Sal B‐treated chondrocytes (Fig. 6A). However, neither the other key mediators in the canonical Wnt signalling pathway, such as *Axin‐1*,* Dvl‐1* and *Gsk‐3β*, nor the hypoxia inducible factor *HIf‐1α* in cultured chondrocytes was affected by the Sal B treatment (Fig. 6A). Further studies demonstrated a similar dosage‐affected fashion of expression pattern for genes coding for SOX9, CYTL‐1, COL II and ACAN in chondrocytes treated with serial diluted Sal B with peaks (*P* < 0.01) at 14.0 μM for *Sox9*,* Col2‐α1* and *Acan*, and 7.0 μM for *Cytl‐1* (Fig. 6B). The cultured chondrocytes were treated with 28.0 μM Sal B for 6, 12, 24 and 48 hrs, and the gene expression patterns showed that *Col1‐α1* remained unchanged accept a significantly increase at 24 hrs at low level (Fig. 6C, left), while the *Col2‐α1* was significantly increased (*P* < 0.01) at all four time points examined at high levels in the treated chondrocytes (Fig. 6C, middle) and that a significantly (*P* < 0.05) increased expression of *Acan* was evidenced at various time points except 12 hrs (Fig. 6C, right). The expression of *Cytl‐1* in cultured chondrocytes was not affected by Sal B in the early hours, became significantly upregulated at 24 hrs onward (Fig. 6D, left) and *IL‐1β* gene, the upregulated inflammatory cytokine in passaged chondrocytes, was only transcribed in early hours (6 hrs) at a significant (*P* < 0.01) high level (Fig. 6D, right).

### The regulatory role of CYTL‐1 on gene transcription in chondrocytes

**Figure 7:**
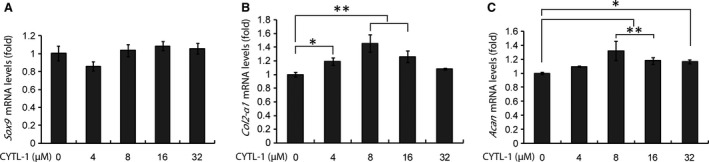
Regulatory action of CYTL‐1 protein on gene expression of *Sox9*,* Col2‐α1* and *Acan* in cultured chondrocytes. Cells were grown in DMEM‐F12 medium containing series diluted hrCYTL‐1 protein and used in cDNA template synthesis for qRT‐PCR analyses. (**A**) *Sox9* expression in chondrocytes was not affected by CYTL‐1. (**B**) *Col2‐α1* expression was significantly increased by treatments with 4.0, 8.0 and 16.0 μM of CYTL‐1 protein. (**C**) *Acan* expression was significantly increased by 8.0 and 16.0 μM, and a less significant increment by a higher concentration (32.0 μM) of the protein. The house‐keeping gene GAPDH was used as an internal reference. *P < 0.05 and **P < 0.01.

Chondrocytes treated with rhCYTL‐1 only showed that the recombinant protein upregulated the expression of genes coding for COL II with a significant increment in the tested concentrations of 4.0 (*P* < 0.05), 8.0 and 16.0 μM (*P* < 0.01), peaked at 8.0 μM; then, the increment became non‐significant at 32.0 μM compared to untreated cells as analysed by qRT‐PCR (Fig. 7B). Similarly, increased transcription of *Acan* was also detected in rhCYTL‐1 treated cells in the range of dilution, peaked at 8.0 μM (*P* < 0.01) with significantly high at 16.0 μM (*P* < 0.01) and 32.0 μM (*P* < 0.05) (Fig. 7C). The rhCYTL‐1 had no effect on the expression of *Sox9* (Fig. 7A).

### MTS assay for chondrocyte proliferation

**Figure 8:**
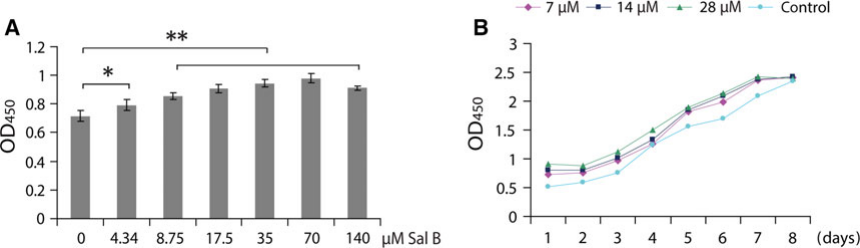
Viability assay of chondrocytes treated with Sal B by MTS. (**A**) Chondrocytes were treated for 24 hrs in culture. Viable cells were significantly increased by 4.34 μM of Sal B and by all other concentrations of Sal B with a highly statistical significance. (**B**) Growth curves were plotted using viabilities (OD_450_) of MTS assays. Increased viable cells by Sal B of all three selected concentrations were maintained up to day 7 in culture and reached stationary phase by day 8. The differences between dilutions of Sal B and at various time points were statistically significant (**). *P < 0.05 and **P < 0.01.

Chondrocytes cultured with series diluted Sal B showed significant increment (*P* < 0.05 for the most diluted [4.34 μM] and *P* < 0.01 for the rests [8.75, 17.5, 35.0, 70.0 and 140.0 μM]) in cell viability (OD_450_ values) as measured by MTS in a dosage‐dependent manner up to the concentration of 70.0 μM, then decreased (Fig. 8A). All three selected Sal B concentrations of 7.0, 14.0 and 28.0 μM enhanced the chondrocyte viability between day 1 and day 7 of the experimental period, then reached the stationary phase by day 8 (Fig. 8B). Differences among these treatments at various time points were statistically significant (*P* < 0.01) with a tendency of significant differences up to day 7 by two‐factor repeated‐measure analyses.

## Discussion

Large numbers of chondrocytes obtained by *in vitro* cultivation of cells isolated from cartilage with low cellularity of limited biopsy are essential to tissue engineering for cartilage reconstructive therapies, such as ACI 1, 3, which usually need at least four passages 4. This study investigated effects of Sal B on passage 3/4 (P3/4) chondrocytes resembling those cultured cells for therapeutic applications. The P3/4 chondrocytes are successfully employed in clarification of regulatory role of factors on cellular functions in various cell models 39, 40, 41. However, accumulated evidence showed that passaged chondrocytes change their gene expression profiles, including matrix proteins, cytokines, matrix proteases and inhibitors, and signalling molecules 42, and dedifferentiate into a more fibroblastic state 43, 44 typically with decreased COL II and ACAN accompanied by increased COL I 4, 6, 7. The activities of cells maintained *in vitro* are ultimately under the influence of culture media, which are specifically formulated and include bioactive stimulants to regulate the expression of genes encoding cytokines and growth factors in the cells for designated cellular properties. Despite the unavoidable dedifferentiation of expanded chondrocytes *in vitro*, the chondrocytic phenotype may either be retained experimentally 45 by optimizing culture conditions or be reversed from dedifferentiated cells by redifferentiation introduced by complemented growth factors 46. The P3/4 chondrocytes cultivated in this study showed typical morphology of colonies (Figs 1A and 2), and they expressed the ECM protein ACAN (Fig. 1C) and both COL II and COL I (Fig. 1B and D), suggesting certain levels of dedifferentiation. The COL II staining was much stronger than COL I, which was further supported by Western blot analysis (Fig. 1E) that showed about three times COL II over COL I (Fig. 1F). The passaged chondrocytes manifested a lesser extent of dedifferentiation than that of previous studies.

The anabolism of cultured chondrocytes is improved by Sal B treatment as evidenced in cellular morphology of microscopy. The pharmacological and regulatory effects of SM and its isolated individual components on various cell types have been widely reported previously and sampled in the Introduction section. This study showed that Sal B, the water soluble component of SM, acted on cultured chondrocytes to promote the anabolic synthesis facilitating cell survival and growth. The Sal B‐treated cells formed more active colonies of thick cell layer and dense cell aggregates (Fig. 2D–F). The promoted nucleic acids synthesis at various cycling stages and showed by different formats (Fig. 3A–H) in treated chondrocytes was illustrated using double‐labelling of DNA and RNA 47 of the genetic basis for cell growth and development, which also revealed a significantly higher level of DNAs and RNAs in treated chondrocytes than that in untreated cells (Fig. 3I). The mitochondria is ‘power station’ of the cell and vulnerable to damage that causes apoptosis 48 and the mitochondrial membrane potential in living cells is detected using rhodamine staining 49. Reduction of the mitochondrial membrane potential, together with enzymatic activities, was observed in chondrocytes of osteoarthritis 50. The mitochondrial membrane potential faded away in living chondrocytes in culture, but was stabilized in the Sal B‐treated cells (Fig. 4). The enhanced mitochondrial function as a power generator and a pivotal player in biological processes of signalling and cellular activities would certainly promote the cellular anabolism. Indeed, the cellular protein expression was stimulated by Sal B in a dosage effective fashion as demonstrated by total protein analysis using BCA assays (Fig. 5A). The major chondrocyte phenotype protein COL II (Fig. 5C) and the key Wnt signalling molecule β‐catenin (Fig. 5B) important for cell fate determination and development were elevated in Sal B‐treated chondrocytes. The expression of genes involved in signal transduction and chondrocyte metabolism was also upregulated (Fig. 6). These results lend molecular supports to the morphological illustrations.

The expression of genes coding for signalling regulators was modulated by Sal B in chondrocytes cultured in monolayer. It has been established that the canonical Wnt/β‐catenin signalling pathway is heavily involved in the activation of gene transcription in chondrocytes 12, 13, 14. The β‐catenin, the central regulator of the Wnt signalling pathway, interacts with hypoxia inducible factor‐1 (HIF‐1α) to activate gene transcription in hypoxic conditions 51, 52. The stabilization and accumulation of cellular β‐catenin protein is believed to be a result of coordinated regulation of a group of signalling mediators/regulators 12, 13, 14, including *Axin‐1*,* Dvl‐1*,* Gsk‐3β* and *Hif‐1α*. This study had shown an elevated β‐catenin protein level in Sal B‐treated chondrocytes (Fig. 5B), but none of these factors were influenced by Sal B (Fig. 6A), suggesting that Sal B increases β‐catenin level possibly not mediated by these Wnt signalling regulators. Neither the β‐catenin level is modulated by the HIF‐1/β‐catenin interaction pathway 51, 52 as *Hif‐1α* remained unchanged (Fig. 6A) in treated cells. The nuclear β‐catenin initiates the formation of downstream transcription complexes and activates gene expression. Sal B stimulated the expression of *Sox9* and *Cytl‐1* genes (Fig. 6), and CYTL‐1 promotes SOX9 activity 19, which generate a peradventure of that the elevated *Sox9* expression may be resulted from increased *Cytl‐1* by Sal B in the treated chondrocytes. In fact, CYTL‐1 did not affect the *Sox9* expression (Fig. 7A), while it increased the expression of *Col2‐α1* and *Acan* (Fig. 7B and C), indicating that Sal B was responsible for upregulating *Sox9* expression rather depending upon *Cytl‐1*. Sal B‐stimulated cytokine CYTL‐1 played a role in the enhancement of the chondrocytic phenotype in passaged cells, but not mediated by SOX9.

Sal B enhanced the expression of downstream genes coding for chondrocytic proteins in cultured chondrocytes. The expression of *Col2‐α1* and *Acan* was upregulated in Sal B‐treated chondrocytes (Fig. 6A) and remained upregulated up to 48 hrs (Fig. 6C). The expression of *Col1‐α1*, the marker molecule of chondrocyte dedifferentiation, was hardly upregulated in the tested cells (Fig. 6C) even six times concentrated cDNA templates (compared to *Col2‐α1* and *Acan*) were used in qRT‐PCRs for resolving 2^−ΔΔCT^ values of *Col1‐α1* amplicons, indicating its low level of transcription. The expression pattern of upregulated differentiation markers of *Col2‐α1* and *Acan*, and unaffected dedifferentiation marker *Col1‐α1* in the Sal B‐treated chondrocytes in culture is discrepant from previously reported expression profile, which suggests downregulated *Col2‐α1* and *Acan* and upregulated *Col1‐α1* in passaged chondrocytes 5, 6, 7. The gene expression pattern was further supported by a low COL I protein level (Fig. 5D) in cultured chondrocytes and remained unchanged, while the COL II was highly increased by Sal B treatment (Fig. 5D). These results suggest that both the formulated culture system and Sal B treatment attributed to the promoted cell viability and ameliorated dedifferentiation status. It is worth noting that the expression of target genes, such as *Col1‐α1*,* Col2‐α1* and *Acan*, responses to the Sal B treatment slower than cytokine *IL‐1β*, which is upregulated in the cultured chondrocytes and induce dedifferentiation by interacting with multiple signalling pathways 53, but the chondrocytic cytokine‐like factor *Cytl‐1* responded to the treatment slowly as other targets (Fig. 6D). After all, the expression of key signalling molecule β‐catenin, the master transcription factors *Sox9* and paracrine cytokine *Cytl‐1* was upregulated by Sal B in cultured chondrocytes, which synthesized significantly higher level of chondrocytic target molecules than control cells, suggesting enhanced anabolic activities. The expression pattern of a highly upregulated *Col2‐α1* and *Acan* accompanied by an unaffected *Col1‐α1* suggests shifts from dedifferentiation to redifferentiation in the Sal B‐treated chondrocytes cultured in monolayer.

Numbers of viable chondrocytes were increased by Sal B treatment in monolayer culture. The promoting effects of Sal B on cell growth have been shown in variety of cells 33, 34. This study found evidence of stimulating effects of Sal B on cellular activities in cultured chondrocytes, including enhanced morphology of cell colonies and aggregates, stimulated synthesis of cellular genetic materials and stabilized mitochondrial membrane potential and upregulated expression of genes coding for important regulatory factors and specific cytokine for chondrocyte proliferation and differentiation and the downstream genes encoding the proteins for chondrocyte growth and phenotype. The promoting actions collectively resulted in significantly increased numbers of viable cells cultured in the given medium in a dosage‐dependent manner (Fig. 8A), and the increments were maintained for up to 7 days in three selected concentrations of 7.0, 14.0 and 28.0 μM, and reached stationary phase by day 8 (Fig. 8B). This *in vitro* culture system using Sal B is capable of generating large numbers of chondrocytes with enhanced cellular, molecular and redifferentiation properties.

The advanced articular cartilage repair treatments, including microfracture, osteochondral grafting, cell implantation and tissue engineering, Largely reply on the activity of chondrocytes. This report provides evidence of stimulating effects of Sal B and CYTL‐1 on cellular activities and gene expression in chondrocytes cultured in monolayer, suggesting their potential application as complementary agents in culture medium for *ex vivo* generation of large numbers of chondrocytes with more differentiation status beneficial to cell‐based therapies of cartilage repair.

## Conflict of interest

The authors confirm that there are no conflicts of interest.

## Author contributions

XY, SJL and SC conceived and designed experiments; SML, PW, WZ, PL and JT prepared samples and performed experiments; XY, SJL, SML and SC analysed data; XY, SJL and SC wrote, and SC made revisions of the manuscript. All authors approved the submitted version of the manuscript.
